# Unlocking the Potential of Three Yam Species (*Dioscorea* spp.) as Starch Sources: Physicochemical, Functional, Morphological, Thermal, and Rheological Characterization

**DOI:** 10.3390/polym18192433

**Published:** 2026-10-06

**Authors:** Katherine Paternina-Sierra, Piedad Montero-Castillo, Diofanor Acevedo-Correa

**Affiliations:** 1 Engineering, Business Administration Program, Agricultural and Agroindustrial Innovation and Development Research Group, Faculty of Economics, University of Cartagena, Cartagena de Indias 130015, Bolívar, Colombia; 2 Agricultural and Agroindustrial Innovation and Development Research Group, University of Cartagena, Cartagena de Indias 130015, Bolívar, Colombia; pmonteroc@unicartagena.edu.co; 3 Tourism Administration Program, Innovation, Management and Engineering Research Group, University of Cartagena, Cartagena de Indias 130015, Bolívar, Colombia; dacevedoc1@unicartagena.edu.co

**Keywords:** *Dioscorea* starch, functional properties, rheology, amylose content, differential scanning calorimetry, adhesion properties

## Abstract

Yams (*Dioscorea* spp.) are tubers with high agro-industrial potential due to their elevated starch content; however, their use remains mainly limited to fresh consumption. This study aimed to comparatively evaluate the physicochemical, functional, morphological, thermal, and rheological properties of native starches from three yam species, *Dioscorea alata*, *Dioscorea rotundata*, and *Dioscorea cayenensis*, to study their potential as sources of starch for food applications. The starches exhibited spherical and ovoid granules. Amylose content was relatively high, at 37.76%, 38.71%, and 32.58% for *D. alata*, *D. rotundata*, and *D. cayenensis*, respectively, exceeding the range commonly reported for yam starches. Significant differences were observed in water and oil retention capacity and swelling power, with *D. cayenensis* showing greater swelling at high temperatures. Pasting properties differed among species; *D. rotundata* exhibited the highest peak viscosity and greater paste stability, while *D. alata* showed the highest pasting temperature and breakdown viscosity. *D. rotundata* also presented the highest final viscosity, whereas setback viscosity did not differ significantly among species. Gelatinization temperatures exceeded 70 °C. All starches displayed pseudoplastic behavior and predominantly elastic properties (G′ > G″), with *D. rotundata* exhibiting the highest yield stress (τ_0_) and greater structural rigidity, as indicated by its higher G′ and G″ values. Overall, these findings highlight the potential of starches from the three yam species as value-added starch sources for food applications, based on their distinct physicochemical, functional, thermal, and rheological properties.

## 1. Introduction

Currently, root and tuber crops constitute the second most important group of cultivated plants worldwide, after cereals [1]. Within this group, yams (*Dioscorea* spp.) stand out for their nutritional, socioeconomic, and agroindustrial importance, as they are one of the most significant food security crops for numerous countries in tropical and subtropical regions [2,3,4], in addition to being of great significance for income generation among small and medium-scale producers [5,6] through their integration into family farming production systems [4,7].

Globally, the most commercially recognized, economically significant, and widely cultivated yam species are *Dioscorea alata* and *Dioscorea rotundata* [4,6,8]. In contrast, the yellow yam (*Dioscorea cayenensis*) does not enjoy the same widespread cultivation or commercial recognition. In Colombia, yam production increased from 47,522 tons in 2015 to 57,953 tons in 2023 [9]. However, in the country, this tuber faces limitations in its production chain, related to low technological development for postharvest handling and agro-industrialization. In this regard, processing yams into flours and starches represents an alternative for adding commercial value and expanding their use in the food sector [10,11,12].

Starch is particularly important for its properties as a stabilizer, thickener, and gelling agent, as well as its ability to improve the performance and texture of foods [7,13]. However, although the main sources of natural starch are cereals such as corn and rice, and tubers such as potatoes and cassava [14], yams (60–89% dry weight starch) represent an unconventional and still underutilized source with great potential [15,16,17]. This is particularly relevant considering that, in 2020–2022, global starch production ranged between 88 and 98 million tons, of which approximately 75% came from corn, followed by cassava (14%), wheat (7%), and potatoes (4%) [18,19], increasing demand throughout the production chain. In light of this, the extraction and characterization of starch from various non-conventional sources, such as the yams *D. alata*, *D. rotundata*, and *D. cayenensis*, represent an alternative to conventional starches.

Currently, unconventional and underutilized sources of starch represent a field of research of growing interest, particularly in the context of food security and sustainable production [20,21,22]. In this regard, the proper utilization of these starches requires a thorough understanding of their physicochemical and functional properties, which are primarily determined by factors such as granule size and morphology, the amylose-to-amylopectin ratio, the degree of crystallinity, and the crystalline structure [8]. These characteristics, influenced by both the biological origin of the starch and the environmental conditions during the development of the botanical source, directly determine its technological behavior and, consequently, its potential applications in the food industry [8,23,24].

However, despite the available information on specific characteristics of yam starches in the region, comprehensive comparative information on the physicochemical composition and thermal, techno-functional, morphological, and rheological properties of starches from *D. alata*, *D. rotundata*, and *D. cayenensis* cultivated in the department of Bolívar (Colombia) remains limited. Based on the known influence of starch composition on its technological behavior, we hypothesized that the three yam species would exhibit significant differences in their physicochemical, techno-functional, pasting, thermal, and rheological properties, which would be associated, at least in part, with differences in their amylose-to-amylopectin ratio. In this context, the objective of this research was to conduct a comparative study of the morphological, physicochemical, thermal, pasting, techno-functional, and rheological properties of native starches from three yam species (*D. alata*, *D. rotundata*, and *D. cayenensis*), to expand knowledge about starches derived from underutilized alternative sources. It is important to note that the findings obtained can guide producers, researchers, and ingredient developers in different regions of the world where yams are cultivated, bearing in mind that the starch properties of a single species may vary depending on the agroecological conditions of each area.

## 2. Materials and Methods

### 2.1. Preparation of the Yam Tubers

The tubers of *Dioscorea alata*, *D. rotundata*, and *D. cayenensis* were obtained from local farmers affiliated with the ASOPROTRACOSAN producers’ association in San Cayetano and were cultivated in San Juan Nepomuceno, Bolívar, Colombia (9.9639° N, 75.1516° W). The tubers were harvested at their optimal physiological maturity, determined based on the complete drying of the aerial parts and the natural detachment of the foliage, which are recognized harvest indicators for *Dioscorea* spp. [25]. Because the tubers were obtained directly from local smallholder farmers through the producers’ association, information regarding individual farms, production lots, or individual plants was not available. Tubers without visible external defects were selected, thoroughly washed to remove soil and adhering impurities, and subsequently rinsed with distilled water to remove residual contaminants.

### 2.2. Starch Isolation

Starch extraction was performed based on the method reported by Zhang et al. [26], with modifications to the extraction conditions. Briefly, the yam tubers were peeled and cut into small pieces and mixed with water at a ratio of 1:8 (*w*/*w*). The mixture was blended for approximately 30 s at constant speed and then filtered through a muslin cloth to remove fibrous material. The filtrate was allowed to stand at 4 °C for 12 h to facilitate starch sedimentation. The supernatant was carefully removed, and the sediment was washed with distilled water. The recovered starch was spread on trays and dried in a forced-air oven (Tornado, Cali, Colombia) at 50 °C until the moisture content was approximately 10%. The dried starch was subsequently ground, passed through a 100 µm sieve, and stored in airtight containers protected from light until further analysis.

### 2.3. Morphological Properties of Starch Granules

#### 2.3.1. Scanning Electron Microscopy (SEM)

The procedure reported by Ochoa and Osorio-Tabón [7] was used with some modifications. The morphology of the samples was analyzed using a scanning electron microscope (JSM-6490LV, JEOL Ltd., Akishima, Tokyo, Japan). Prior to observation, the samples were mounted on aluminum holders using double-sided conductive carbon tape and subsequently coated with a thin 15-nm layer of gold to obtain conductive samples. Micrographs were taken using an acceleration voltage of 20 kV and magnifications of 250, 500, and 2000×.

#### 2.3.2. Particle Size Distribution

The particle size distribution of the starches was determined at room temperature using a laser diffraction particle size analyzer (Mastersizer 2000, Malvern Instruments Ltd., Malvern, Worcestershire, UK), equipped with a Hydro 2000MU dispersion unit. Measurements were performed using water as the dispersant, with refractive indices of 1.59 for starch and 1.33 for water. Approximately 0.015% (*v*/*v*) of the starch suspension was dispersed under constant agitation with a stirrer at 2500 rpm. A general (spherical) optical model was applied, with a particle size detection range between 0.02 µm and 2000 µm. The results are expressed in terms of volume distribution, characterized by the values D [10], D [50], and D [90], which represent the diameters below which 10%, 50%, and 90% of the total sample volume are found, respectively. In addition, the surface-weighted (D [3;2]) and volume-weighted (D [4;3]) mean diameters are reported [17].

### 2.4. Physicochemical Characterization of Yam Starch

#### 2.4.1. Determination of pH and Acidity

The pH of the samples was measured using a Hanna Instruments pH meter, which was previously calibrated with buffer solutions at pH 4, 7, and 10, following the method described by Ngoma et al. [27]. Acidity was determined by volumetric titration according to the AOAC 942.15 method [28]. For this, a 0.1 N NaOH solution was used along with 1% phenolphthalein as indicator, and the volume was recorded until a slight pink color appeared.

(1) $$\%\;Acidity=\frac{GxNx\;meq\;of\;acid}{M}X\;100$$

where:

G: volume of NaOH consumed; N: normality of the solution; meq of acid: mL of equivalent acid; M: g of sample.

#### 2.4.2. Starch Color Determination

The color of the samples was evaluated using a colorimeter (Konica Minolta, Sakai, Osaka, Japan), following the methods reported by Kimbonguila et al. [29] and Setyawan et al. [30]. Before taking the measurements, the equipment was calibrated using the CIELAB system (L*, a*, b*), which is commonly used to assess color in food samples. The whiteness index (WI) was subsequently calculated using the following equation:

(2) $$\%WI\;=\;100-\sqrt{{\left(100\;-\;L*\right)}^{2}\;+({a*)}^{2}\;+({b*)}^{2}}$$

#### 2.4.3. Proximate Analysis of Starch

For food quality characterization, the official AOAC [28] methods were followed: moisture content (method 925.10), crude protein (method 920.87), crude fat (method 920.39), ash (method 923.03), and crude fiber (method 920.86). Carbohydrate content was determined by difference.

#### 2.4.4. Total Amylose and Amylopectin Content

The amylose content was determined by iodine adsorption spectrophotometry following the method reported by Chiranthika et al. [23], based on the formation of a blue-colored complex between amylose and an iodine–iodide solution. Briefly, 10 mg of the starch sample was weighed, and 10 mL of dimethyl sulfoxide (DMSO; Sigma-Aldrich, St. Louis, MO, USA) was added. The mixture was heated in a water bath at 80 °C for 15 min in the dark. Subsequently, 100 μL of the sample solution was transferred and diluted with 1.8 mL of distilled water and 100 μL of iodine–iodide solution, prepared with 0.2 g of iodine (I_2_; Sigma-Aldrich, St. Louis, MO, USA) and 2 g of potassium iodide (KI; Sigma-Aldrich, St. Louis, MO, USA) in 100 mL of distilled water. After standing for 20 min in the dark, the absorbance was measured at 620 nm. The amylose content was quantified using a standard calibration curve, and the results are expressed as a percentage of the starch sample. Amylopectin content was calculated by difference as 100 minus the amylose content.

### 2.5. Functional Properties of Starch

#### 2.5.1. Water-Holding Capacity (WHC) and Oil-Holding Capacity (OHC)

The WHC was determined by adding 10 mL of distilled water to 0.5 g of sample at room temperature. The mixture was subsequently incubated in a water bath at 60 °C for 30 min, followed by centrifugation at 3000 rpm for 15 min. After centrifugation, the supernatant was removed, and the tubes containing the sediment were weighed. Results are expressed as grams of water retained per gram of starch [31].

(3) $$WHC\;(\frac{g}{g})=\frac{\left(Weight\;of\;the\;tube\;with\;sediment-weight\;of\;the\;empty\;tube\right)-Weight\;of\;the\;sample}{Weight\;of\;the\;sample}$$

For the determination of OHC, 0.5 g of sample was placed in a pre-weighed centrifuge tube, and 6 mL of sunflower oil (density: 0.918 g/mL) was added. The mixture was kept at 25 °C for 30 min and then centrifuged at 5000 rpm for 30 min. After removing the unabsorbed oil, the tubes were weighed again, and the OHC was calculated based on the retained oil [2].

(4) $$OHC\;\left(\frac{mL}{g}\right)=\frac{Weight\;of\;tube\;with\;sediment-weight\;of\;sample-weight\;of\;empty\;tube/d}{Weight\;of\;sample}$$

#### 2.5.2. Swelling Power (SP) and Solubility (SO)

The sample was dispersed in distilled water at a 1:10 ratio (*w*/*v*) in a centrifuge tube and subjected to thermal treatment in a water bath at 55, 65, 75, 85, and 95 °C for 30 min under continuous stirring. After heating, the samples were left to cool down to room temperature and then centrifuged at 6000 rpm for 15 min. The supernatant was carefully collected and transferred to evaporation dishes, where it was dried at 110 °C for 3 h. After drying, the residue (representing the soluble fraction) and the remaining insoluble material were weighed to evaluate the degree of solubilization [2,32].

(5) $$SP=\frac{Sediment\;weight}{Sample\;weight-soluble\;weight}$$

(6) $$SO=\frac{Soluble\;weight}{Sample\;weight}\;X100$$

### 2.6. Thermal Properties of Starch

#### 2.6.1. Differential Scanning Calorimeter (DSC)

The thermal properties of the yam starches were evaluated using a differential scanning calorimeter (Discovery DSC, TA Instruments, New Castle, DE, USA), previously calibrated with 99.99% pure indium (melting peak temperature, T_p_ = 156.6 °C; ΔH = 28.56 J/g). For each analysis, 3 mg of starch sample and 7 mg of deionized water were weighed into hermetically sealed aluminum DSC pans, corresponding to a water-to-starch ratio of approximately 2.33:1 (*w*/*w*). The sealed pans were equilibrated at room temperature for 4 h before analysis. An empty sealed pan was used as the reference. The samples were heated from 20 to 140 °C at a rate of 10 °C/min. The onset (T_o_), peak (T_p_), and conclusion (T_c_) temperatures and the enthalpy change (ΔH) associated with the thermal transition were determined from the DSC thermograms. The gelatinization enthalpy (ΔH) was calculated from the area of the endothermic peak and is expressed in J/g. The peak temperature (T_p_) was considered the gelatinization temperature of the starch samples [26].

#### 2.6.2. Thermogravimetric Analysis

The thermal stability of the starch samples was assessed using a thermogravimetric analyzer (TGA Q500, TA Instruments, New Castle, DE, USA). Approximately 13 mg of each sample was used for the analysis. The measurements were carried out from room temperature up to 600 °C at a heating rate of 10 °C/min. An inert atmosphere was maintained throughout the experiment by supplying a continuous nitrogen flow of 100 mL/min, in order to avoid thermo-oxidative degradation [8].

### 2.7. Rheological Analysis

The rheological properties of yam starch suspensions were evaluated using a rotational rheometer (HR 10 Discovery, TA Instruments), following Argaw et al. [33]. A 5% (*w*/*v*) suspension was prepared in distilled water, heated at 90 °C for 7 min, and cooled to room temperature. Measurements were carried out using a 40 mm parallel plate geometry with a 1 mm gap. Flow curves were obtained at 25 °C over a shear rate range of 1 to 1000 s^−1^ and fitted to the Herschel–Bulkley model. Additionally, oscillatory tests were performed to determine the storage (G′) and loss (G″) moduli within a frequency range of 0.1 to 100 rad/s at a constant strain of 0.2%.

### 2.8. Starch Pasting Properties

The pasting properties were determined using a Discovery HR 10 Hybrid Rheometer (TA Instruments, New Castle, DE, USA) equipped with a 40-mm-diameter smooth geometry, a Peltier plate, and a 1000 μm gap. Starch suspensions (8% *w*/*v*) were initially held at 50 °C for 1 min, followed by heating to 95 °C at a rate of 6 °C/min over 7.5 min and holding at 95 °C for 5 min. The samples were subsequently cooled to 50 °C at 6 °C/min over 7.5 min and held at 50 °C for 2 min. The mixing speed was maintained at 80 rpm. The measurements were performed in triplicate. The pasting temperature, peak viscosity, breakdown viscosity, setback viscosity, and final viscosity were determined according to Dar et al. [34].

### 2.9. Data Analysis

All data were analyzed using Statgraphics Centurion software versión 16.2.04 (Statgraphics Technologies, Inc., Warrenton, VA, EE. UU.). Three independent replicates were used for each analysis, and the results are expressed as mean ± standard deviation. Analysis of variance (ANOVA) was performed to determine significant differences among yam starches, and means were compared using Tukey’s multiple comparison test at a 95% confidence level (*p* < 0.05).

## 3. Results and Discussion

### 3.1. Morphological Properties

#### 3.1.1. Scanning Electron Microscopy (SEM)

**Figure 1:**
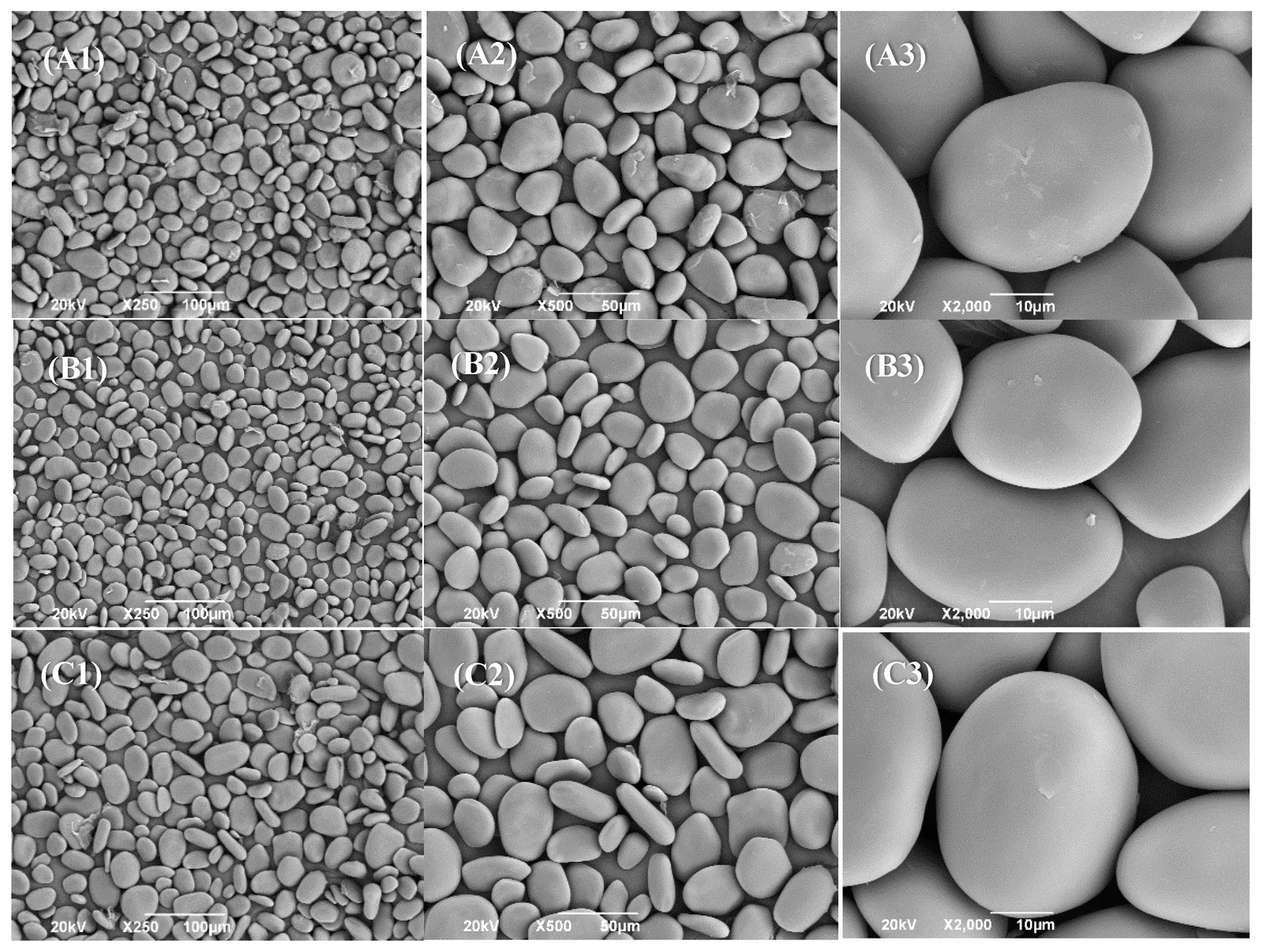
SEM micrographs of starch from: *Dioscorea alata* at 250× (**A1**), 500× (**A2**), and 2000× (**A3**); *Dioscorea rotundata* at 250× (**B1**), 500× (**B2**), and 2000× (**B3**); *Dioscorea cayenensis* at 250× (**C1**), 500× (**C2**), and 2000× (**C3**).

SEM images of the three yam starches analyzed are shown in Figure 1. The granule shapes in *D. alata*, *D. rotundata*, and *D. cayenensis* were similar, predominantly oval, ellipsoidal, and rounded, with some minor variations in shape. All exhibited smooth surfaces with no evidence of cracking or fissures. In this regard, granule morphology showed minimal variations among the three species, being similar to that described for most yam species [7,25,35,36,37,38,39].

It should be noted that the yam starch granules from *D. rotundata* and *D. cayenensis* (Figure 1(B1–B3,C1–C3)) exhibited smoother surfaces, whereas some layered structures were observed on the surfaces of *D. alata* starch granules (Figure 1(A1–A3)), possibly associated with proteins not removed during the isolation process. This observation is consistent with the higher protein content found in *D. alata* starch (0.96 ± 0.09%), compared with *D. rotundata* (0.46 ± 0.05%) and *D. cayenensis* (0.37 ± 0.05%) (Table 3). The presence of residual protein may influence starch–water interactions and may therefore contribute to differences in the techno-functional properties of the starches [40].

For *D. alata* starches, studies such as those by Chiranthika et al. [23], Ochoa & Osorio-Tobón [7], Olumide Fawale et al. [41], Tanimola et al. [16], and Oliveira et al. [13] reported scanning electron microscope images of *D. alata* starch, where the granule shapes are similar to those in the present study. In the case of *D. rotundata* starch granules, the results coincide with those described by Tanimola et al. [16] and Martinez Villadiego et al. [8]. Regarding *D. cayenensis*, although available studies are more limited, the observed starch granules show similarity to those reported by Otegbayo et al. [25].

#### 3.1.2. Particle Size Distribution

**Figure 2:**
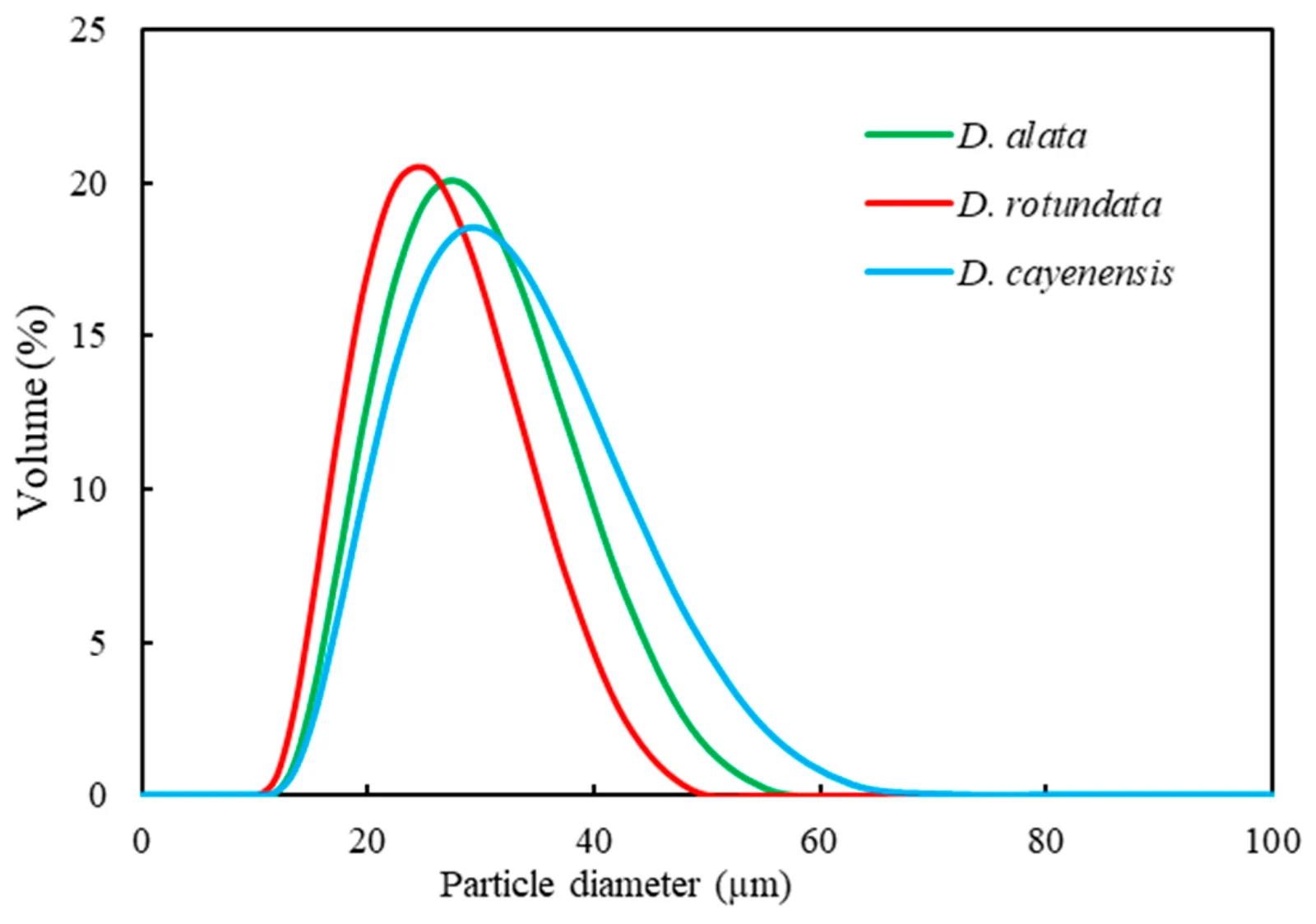
Particle size distribution of yam starches.

The particle size distribution of the yam starches is shown in Figure 2 and Table 1. All starches exhibited a unimodal distribution, which may be associated with their low moisture and protein content, as noted by Liu et al. [35]. The diameter ranges were 10.50–62.76 μm for *D. alata*, 10.50–48.61 μm for *D. rotundata*, and 10.50–71.30 μm for *D. cayenensis*, indicating differences in granule sizes among the studied species. In the case of *D. rotundata*, the curve shifted to the left, indicating a higher proportion of smaller particles compared to *D. alata* and *D. cayenensis*. These results are consistent with those reported by Shao et al. [37], Lu et al. [36], and Martinez Villadiego et al. [8], who highlight that differences in shape and size may occur due to differences between species, as well as biological, biochemical, physicochemical, and environmental factors.

Starch granules can be classified as large (>25 µm), medium (10–25 µm), small (5–10 µm), and very small (<5 µm) [25]; accordingly, on average, the starch granules of these yam species were all large. The average particle size (D [50]) varied significantly among the starches evaluated; *D. cayenensis* starch had the highest value (29.03 μm), followed by *D. alata* (26.98 μm), while *D. rotundata* had the smallest size (24.15 μm). This trend was consistent for the D [10] and D [90] parameters, which represent the sizes below which 10% and 90% of the total volume, respectively, are found, confirming that *D. rotundata* has smaller and more homogeneous granules compared to the other two species. It should be noted that D [50] is the most representative parameter for describing particle size distribution, as it corresponds to the median diameter that divides the granule population into two equivalent fractions [42].

It is worth noting that, according to previous studies such as those conducted by Li et al. [43], Zou et al. [39], and Otegbayo et al. [25], the size of yam starch granules generally ranges from 18 to 50 μm, values that are consistent with those obtained in the present study, whereas that of potato and cassava starch is 15 to 75 μm and 6 to 33 μm, respectively, and the diameter of rice starch granules is 3 to 8 μm, reflecting variations attributable to botanical origin and growing conditions, as mentioned earlier.

Similarly, the mean diameters, D [4;3] and D [3;2], were also significantly smaller in *D. rotundata* compared to the other two starches, reinforcing the evidence that this species is characterized by smaller particles [44]; the values fell within the ranges reported by Ouyang et al. [45] and Ochoa & Osorio-Tobón [7] for each parameter.

These results suggest that the starches from *D. rotundata* have smaller, more compact granules. However, unlike shape, it has been reported that granule size influences functional properties such as swelling capacity, gelatinization, and solubility, as well as dough-forming properties [16,25]. In this regard, variation in granule size has been associated with different shear sensitivity, which could affect the texture of food products made with these starches. Furthermore, research has linked larger granules to greater swelling capacity and higher viscosities [16]. Therefore, granule size is a relevant property for the food and industrial applications of starches. In this regard, the starches from *D. rotundata*, *D. alata*, and *D. cayenensis* can be used in applications requiring good viscosity and swelling capacity [25].

### 3.2. Physicochemical Properties

#### 3.2.1. pH, Titratable Acidity and Color

The pH value of starch is a significant factor, as it influences its thermal and gelatinization properties, as well as its potential applications [46]. As shown in Table 2, the pH analysis revealed values ranging from 6.07 to 7.10, indicating that the three starches evaluated tend toward neutrality. *D. alata* starch had the lowest pH, which was significantly different from those of *D. rotundata* and *D. cayenensis*. These results fall within the ranges reported in the literature. Oliveira et al. [13] reported a pH of 6.69 for *D. alata* starches, while Salgado-Ordosgoitia et al. [14] reported values of 6.2 and 6.8 for *D. alata* and *D. rotundata*, respectively, very similar to those obtained in the present study. These values were also consistent with those reported by Donaldben et al. [47]. Similarly, Otegbayo et al. [25] reported that the pH of yam starches ranges from 6.5 in *D. rotundata* and *D. alata* to 7.8 in *D. cayenensis*.

Regarding titratable acidity, significant differences were observed: the starch from *D. alata* exhibited the highest acidity (0.31), while *D. rotundata* (0.07) and *D. cayenensis* (0.05) showed considerably lower values, which is consistent with the pH values observed for each of these starches. In turn, these values were lower than those reported by Tanimola et al. [16], who noted that in *D. rotundata*, titratable acidity ranged from 0.14 to 1.99, whereas, in *D. alata*, the ranges varied from 0.74 to 2.00. Thus, the pH and titratable acidity ranges suggest that the yam starches studied are a low-acidity product, comparable to taro and cassava starches [46], and therefore tend to exhibit greater chemical stability and a lower risk of acid hydrolysis, which facilitates their storage and use in food matrices without altering the flavor or interfering with processing. For this reason, several authors consider a pH close to neutrality to be a favorable condition for industrial applications [16,46].

Furthermore, regarding instrumental color parameters, statistically significant differences were observed in the lightness value (L*) among the starches of the three species studied. The starch from *D. cayenensis* exhibited the highest lightness, followed by *D. rotundata* and *D. alata*, indicating that the starch from *D. cayenensis* appears visually whiter. This behavior, associated with low chroma values on the a* (−0.76) and b* (3.46) axes, confirms the absence of intense red/green and yellow/blue hues; this is further supported by the higher %WI values obtained for this starch (93.11).

The L* values obtained for the three starches were higher than the ranges described by Tortoe et al. [46] (L*: 80.30–90.20) for *D. alata* starches; however, they were below those reported by Oliveira et al. [13] (L*: 98.12; a*: 0.56) for yam starch. It should be noted that *D. alata* starch exhibited a higher a* value (0.68) compared to that reported by those authors.

Nevertheless, the variations observed in the color parameters, although statistically significant, did not reach a sufficient visual perception threshold to clearly differentiate the species *D. alata*, *D. rotundata*, and *D. cayenensis* based solely on this index. These results are comparable to those of corn starch (L* 98.87; a* −0.45; b* 4.91; %WI 94.95) [48], which is widely used and accepted in the food industry, supporting the potential of yam starches as a viable alternative in this sector.

#### 3.2.2. Proximate Analysis

Table 3 presents the chemical composition of the yam starches. The average content of protein, crude fiber, fat, and ash was less than 1% in all cases. The low content of these non-starch constituents reflects the efficiency of the extraction process and the high purity of the starch obtained [13,39]. Regarding moisture content, the starch from *D. alata* had a significantly higher value compared to *D. rotundata* and *D. cayenensis*; however, in all three species, levels remained below 13%, consistent with reports by Jiang et al. [15], Lu et al. [36], Salgado-Ordosgoitia et al. [14], and Zou et al. [39]. This moisture range is recommended to ensure safe storage [13,46]. In particular, *D. rotundata* is distinguished by its low moisture content [49].

It is important to note that, with regard to ash, fat, and fiber content, the starches from these species did not show statistically significant differences among themselves, which can be explained by the fact that fat and fiber are not major components of the tuber’s physiological composition [14]. In this regard, the fat content in *D. alata* starch was lower than that reported by Salgado-Ordosgoitia et al. [14], Otegbayo et al. [50], and Chiranthika et al. [23] (0.16%, 0.09%, and 0.11%, respectively) and lower for *D. rotundata*, as reported by Salgado-Ordosgoitia et al. [14] (0.33%). The fiber content fell within the range reported by Salgado-Ordosgoitia et al. [14]. Likewise, the uniformity observed in dry matter content among the analyzed samples is noteworthy, consistent with the absence of statistical differences in ash content, whose value was similar to that reported by Chiranthika et al. [23] and Otegbayo et al. [50] (0.19 to 0.32%) for *D. alata*.

In terms of protein content, the starch from *D. alata* exceeded that of the other two species in this study and showed values higher than those reported by Chiranthika et al. [23], Lu et al. [36], and Otegbayo et al. [50], by 0.19%, 0.13%, and 0.07%, respectively, but similar to those reported by Salgado-Ordosgoitia et al. [14] and Oliveira et al. [13] for *D. alata* starch (0.95% and 0.90%, respectively) and lower than those reported by Donaldben et al. [47] (1.65%). However, the values for *D. rotundata* were lower than those obtained by Salgado-Ordosgoitia et al. [14] and Donaldben et al. [47] (1.68% and 1.55%, respectively).

The carbohydrate content consists mainly of starch (amylose and amylopectin), since simple sugars and fiber were removed during the isolation process. Among the three yam species, *D. rotundata* had the highest carbohydrate content, exceeding that reported by Donaldben et al. [47] (86.20%), indicating a higher concentration of pure starch, followed by *D. cayenensis* and *D. alata*; the latter was similar to the content reported by Donaldben et al. [47] (85.59%). This high content makes *D. rotundata* an attractive option for industrial applications where starch extraction is to be maximized. Likewise, *D. cayenensis*, although with a slightly lower content, remains an effective source of carbohydrates and could have a better functional profile due to its high proportion of amylopectin. In this regard, Polycarp et al. [49] state that these two species have higher levels of carbohydrates and energy, along with appreciable amounts of minerals, making them nutritious and reliable as crops for food and energy security. For its part, *D. alata*, with the lowest carbohydrate content, stands out for its higher proportion of amylose and ash, making it suitable for specific applications such as the production of low-glycemic-index foods.

#### 3.2.3. Amylose and Amylopectin Content

Yam starch contains an average of 20–30% amylose and 70–80% amylopectin, classifying it as “normal”, although values differ among species [24]. Thus, starches with less than 10% amylose are classified as “waxy,” while those classified as high in amylose have concentrations exceeding 35% of this component [8]. Based on this information and according to Table 3, the starches of *D. alata* and *D. rotundata* can be considered high in amylose, suggesting that these tubers constitute potential sources of novel starch with high amylose content. Given that a positive correlation has been demonstrated between higher amylose content and higher levels of resistant starch, and consequently lower digestibility [15,51], increasing amylose content could provide functional benefits to consumers by promoting slower carbohydrate digestion [7,52]. In this regard, the starches from *D. alata* and *D. rotundata* yams represent a promising alternative for the development of functional foods.

Regarding the amylose content of *D. alata* starches, the values obtained were above the reported normal range (20–30%) [11,13,50]. The results were comparable to those reported by Chiranthika et al. [23] (35.50) and by Lu et al. [36] for *D. opposita* (37.90). However, they were lower than those described by Jiang et al. [15] for both *D. alata* (40.24%) and *D. opposita* (48.09%). Consistently, Huang et al. [53] also reported high amylose contents in *D. alata* starches (40.1%) from El Carmen de Bolívar (Colombia) [8].

Previous studies conducted by Martinez Villadiego et al. [8] and Salgado-Ordosgoitia et al. [14] reported amylose–amylopectin content in the native starch of *D. rotundata* of 25.59–74.4% and 23.64–76.40%, respectively. In contrast, in the present study, the values obtained for this species were 38.71% amylose and 61.29% amylopectin, indicating a higher proportion of amylose and, consequently, a reduction in amylopectin content. This increase in the amylose fraction is particularly relevant, as it directly influences the functional properties of the starch, favoring the formation of firmer gels and lower rapid digestibility. Furthermore, this starch could represent an advantage in industrial and food applications where greater thermal stability is required. Meanwhile, the amylose content of *D. cayenensis* starch (32.58%) was higher than that reported by Mawoneke et al. [24] (22.06%) and close to that of commercial starches such as corn starch (28.3%) [25].

These differences in amylose and amylopectin content observed among genotypes of the same species and across different species may be attributed to factors such as genetic variations, environmental factors, stage of maturity, and soil type [23,50]. These factors may also contribute to the differences observed between the amylose content of *D. rotundata* in the present study and values reported in previous studies [8,14]. In this regard, it has been documented that genotypes of various yam species exhibit variations in amylose content ranging from 1.4% to 50% [54], which could confer diverse physicochemical and functional properties on the starch, as well as different technological applications. In the present study, *D. rotundata* exhibited the highest amylose content, whereas *D. cayenensis* showed the lowest value, while *D. alata* presented an intermediate amylose content. These differences may contribute to the distinct functional and rheological behavior observed among the starches and highlight the potential of the three yam species as sources of starch with different technological characteristics.

### 3.3. Functional Properties

#### 3.3.1. Water-Holding Capacity (WHC) and Oil-Holding Capacity (OHC)

Starches are primarily composed of amylose and amylopectin, molecules that largely determine their functional properties [55,56,57]. However, other minor components, such as proteins and lipids, can also influence these characteristics. Understanding these properties with a view to their application in food processing is essential for harnessing their potential in the industry. The results obtained are presented in Table 4.

WHC indicates the ability of starch to retain water, both internal and added, when subjected to force, pressure, centrifugation, or heating [23]. This property significantly influences the improvements in food juiciness and texture and, consequently, sensory qualities such as mouthfeel, increased satiety, and food volume [58], aspects relevant to bakery products and food matrices that require hydration to improve their handling properties [11,27,47]. In this study, the WHC of the starches ranged from 1.97 to 2.15 g/g, values higher than those reported by Donaldben et al. [47] (0.868 to 0.915 g/g) for yam starches, but lower than those reported by Tortoe et al. [46] (4.10–4.89 g/g).

The WHC was highest in *D. alata*, followed by *D. rotundata* and *D. cayenensis*. This could be associated with its higher protein content compared to *D. cayenensis* and *D. rotundata*, in agreement with Tortoe et al. [46], who note that the WHC of yam starches is determined by protein–water interaction, such that a higher protein content provides more available binding sites for water molecules. However, although the protein content of *D. rotundata* (0.46%) was slightly higher than that of *D. cayenensis* (0.37%), this difference was not statistically significant, which explains why their WHC values also did not differ significantly from one another. These differences may also be attributed to the higher amylose content in *D. rotundata* and *D. alata* (38.71 and 37.76 g/100 g, respectively), which increases their affinity for water due to the hydrophilic nature of this fraction [1,11]. Likewise, it is possible that in these starches there is less amylose–amylose and amylose–amylopectin interaction, which has been associated with a higher availability of free hydroxyl groups capable of forming bonds with water [10,50].

Differences in WHC can be attributed to variations in the structure and morphology of starch, amylose, and amylopectin, as well as the presence of salts, proteins, and other granular components resulting from differences in genetic composition, cultivation practices, and geographic origin, and the fact that yams are a highly diverse crop [11,24,46].

On the other hand, OHC is primarily associated with the physical entrapment of lipids and partly reflects protein–fat interactions in food formulations [59]. In this study, OHC values ranged from 0.52 to 0.83 g/g, with *D. alata* starch again standing out, consistent with its higher protein content. Added to this effect is the contribution of amylose, since a higher proportion of linear chains favors the formation of networks capable of retaining both water and oil [11]. However, these values were lower than those reported by Donaldben et al. [47] (1.032 to 1.100 g/g). A high OHC improves the retention of lipid compounds and flavor development, which is advantageous in high-fat baked goods [23,24].

#### 3.3.2. Swelling Power (SP) and Solubility (SO)

Table 5 presents the results for the SP and SO of the starches, evaluated in the range of 55 to 95 °C. As expected, both parameters increased progressively with rising temperature due to starch gelatinization [32,60], although with variations among species. These differences were most evident at temperatures above 85 °C, where *D. alata* and *D. cayenensis* exhibited significantly higher values than *D. rotundata*. This trend became more pronounced at 95 °C, with *D. alata* and *D. cayenensis* reaching the highest values for swelling and solubility, while *D. rotundata* remained at a significantly lower level. These results fall within the swelling capacity range previously reported for *D. alata* [11,23,24,46], although they exceed the solubility values reported by those same authors. Similarly, Mawoneke et al. [24], Effah-Manu et al. [11], and Pérez et al. [61] reported similar swelling power ranges for *D. rotundata* starches but with lower solubility values. In contrast, *D. cayenensis* starch exhibited higher swelling power and solubility similar to that reported by Mawoneke et al. [24].

Chemical composition analysis revealed that the starch from *D. rotundata* had the highest amylose content (38.71 g/g) and the lowest amylopectin content (61.29 g/g). The high proportion of amylose has been associated with restricted starch expansion, as it reinforces the internal network of the granule and helps maintain the integrity of the swollen particles [23,24,46], which would explain why it achieved a significantly lower swelling capacity in the range of 85 to 95 °C.

In contrast, *D. cayenensis* had the lowest amylose content (32.58 g/g) and the highest amylopectin content (67.42 g/g). This composition promotes granule expansion; in fact, a positive correlation has been reported between amylopectin content and swelling capacity [23], which was reflected in its highest recorded value at 95 °C (11.04 g/g). Meanwhile, the starch from *D. alata* exhibited intermediate behavior (37.76 g/g of amylose and 62.24 g/g of amylopectin), achieving high swelling capacity and the highest solubility at 95 °C (5.13%), suggesting greater leaching of amylose into the medium, which is consistent with the findings of Bora et al. [32] and Effah-Manu et al. [11], who link a high solubility index to the easy dissolution of amylose weakly bound to the molecular structure and susceptible to leaching during the swelling process. This characteristic is consistent with the higher water-holding capacity observed for this starch, associated with reduced amylose–amylose and amylose–amylopectin interactions [10,50,51].

These results indicate that the amylose/amylopectin ratio plays a decisive role in the functional response of starches to high temperatures [54,61]. While the higher amylopectin content in *D. cayenensis* promoted swelling, the balance in *D. alata* allowed for both high swelling capacity and high solubility. In contrast, the high amylose content in *D. rotundata* restricted granule expansion and reduced solubility, reflecting greater structural stability. In general, some studies indicate that the starches of certain yam varieties have low swelling capacity compared to commercial starches, and thus can be incorporated into food systems where thick porridges are undesirable [24], a behavior associated with a high amylose content in these starches [11,15].

### 3.4. Thermal Properties

#### 3.4.1. Differential Scanning Calorimeter (DSC)

**Figure 3:**
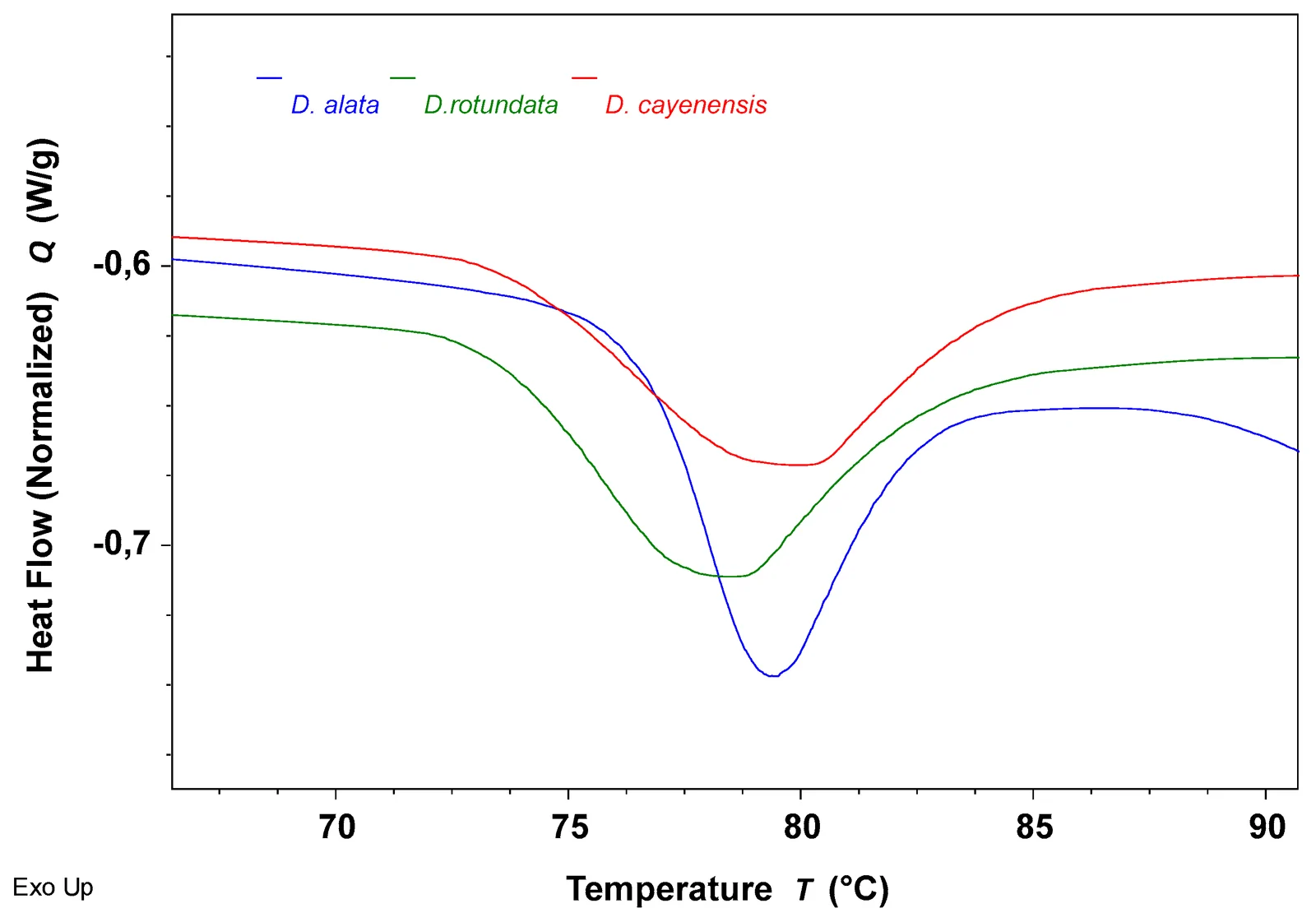
DSC thermogram of yam starches from *D. alata*, *D. rotundata* and *D. cayenensis*.

The DSC results for the starches of *D. alata*, *D. rotundata*, and *D. cayenensis* are presented in Figure 3 and Table 6. The results showed a single endothermic transition associated with gelatinization, a phenomenon corresponding to the breaking of amylopectin double helices and the loss of molecular order in the semicrystalline regions of the granule [52,62,63]. The absence of additional or overlapping peaks indicates that no distinct thermal transitions attributable to amylose–lipid complexes or crystalline fractions with different thermal stability were detected under the experimental conditions used. However, the absence of a distinct peak does not necessarily exclude the presence of amylose–lipid complexes, as such transitions may not be sufficiently resolved under the heating conditions employed. Similar thermal behavior has been reported in some purple *D. alata* starches [36].

The onset temperatures (T_o_) obtained were higher than those reported by Oladebeye et al. [64] for wild yam (*D. villosa*), whose T_o_ values range from 59.21 to 63.12 °C, indicating greater thermal stability of the species evaluated in this study. However, the values were slightly lower than those reported by Zou et al. [39] for some Chinese yam varieties, where T_o_ ranges from 84.12 to 88.59 °C. This difference could be attributed to genetic and environmental factors, as well as variations in the isolation method, as noted by Chiranthika et al. [23].

Regarding peak temperature (T_p_), the values obtained are comparable to those described by Aprianita et al. [65] for Indonesian varieties (73.9 °C). Likewise, they are close to the values reported by Gbassi et al. [66] for *D. rotundata* (69.9–73.4 °C), although slightly higher temperatures were observed in this study, suggesting greater relative crystalline stability. These differences may be related to the molecular structure of amylopectin, crystallinity, and the presence of phosphate bonds within the starch structure [24].

The starch from *D. alata* exhibited the highest onset temperature, while *D. cayenensis* showed the highest final temperature, even exceeding some values reported for *D. alata* in Sri Lanka (74.8–74.9 °C) [67]. This behavior suggests differences in the thermal stability of the crystalline domains and in the structural homogeneity of the granule, considering that the gelatinization range (T_f_–T_o_) is an indicator of structural heterogeneity [64,68].

With regard to the gelatinization enthalpy (ΔH), the values obtained were considerably lower than those reported for other yam starches, where ΔH ranges from 12.15 J/g [69] to 23.08–31.23 J/g [39]. This difference could indicate a lower degree of crystallinity or a lower proportion of organized double helices in the analyzed samples [13]. According to Zou et al. [70], ΔH is directly related to the energy required to disrupt crystalline double helices, so lower values could reflect a partially less ordered granular architecture.

In addition, studies have indicated that factors such as particle size, surface area, and granule homogeneity can influence thermal behavior and the observed ΔH [64,68,71]. Therefore, the lower ΔH values observed in the present study compared with those reported in the international literature may be associated with differences in genetic and environmental variability [23], as well as with differences in the physical characteristics of the starch granules and experimental conditions, particularly the starch-to-water ratio used in the DSC analysis. These factors may affect the organization and stability of the molecular structures involved in gelatinization and, consequently, the energy required for the thermal transition. T_p_ values close to 79 °C place these starches within the typical range reported for tropical tubers, higher than that reported by Alvani et al. [72] for potato starch (59–71 °C) and similar to that reported by Han et al. [69] and Jiang et al. [73] for common corn (T_p_ approximately 72.8 °C).

From a technological standpoint, T_p_ values close to 79 °C indicate medium-to-high thermal stability, which in baking suggests that gelatinization occurs during the later stages of baking, promoting structural stability and gas retention, and contributing to a more uniform crumb. On the other hand, the low gelatinization enthalpy indicates that less energy is required to disrupt the crystalline regions, which may facilitate gelatinization and the interaction of starch with other components of the dough. However, this lower structural stability could also be associated with greater susceptibility to retrogradation during storage [11]. Overall, these starches exhibit thermal behavior suitable for bakery applications, offering a balance between stability during baking and gelatinization capacity.

#### 3.4.2. Thermogravimetric Analysis

**Figure 4:**
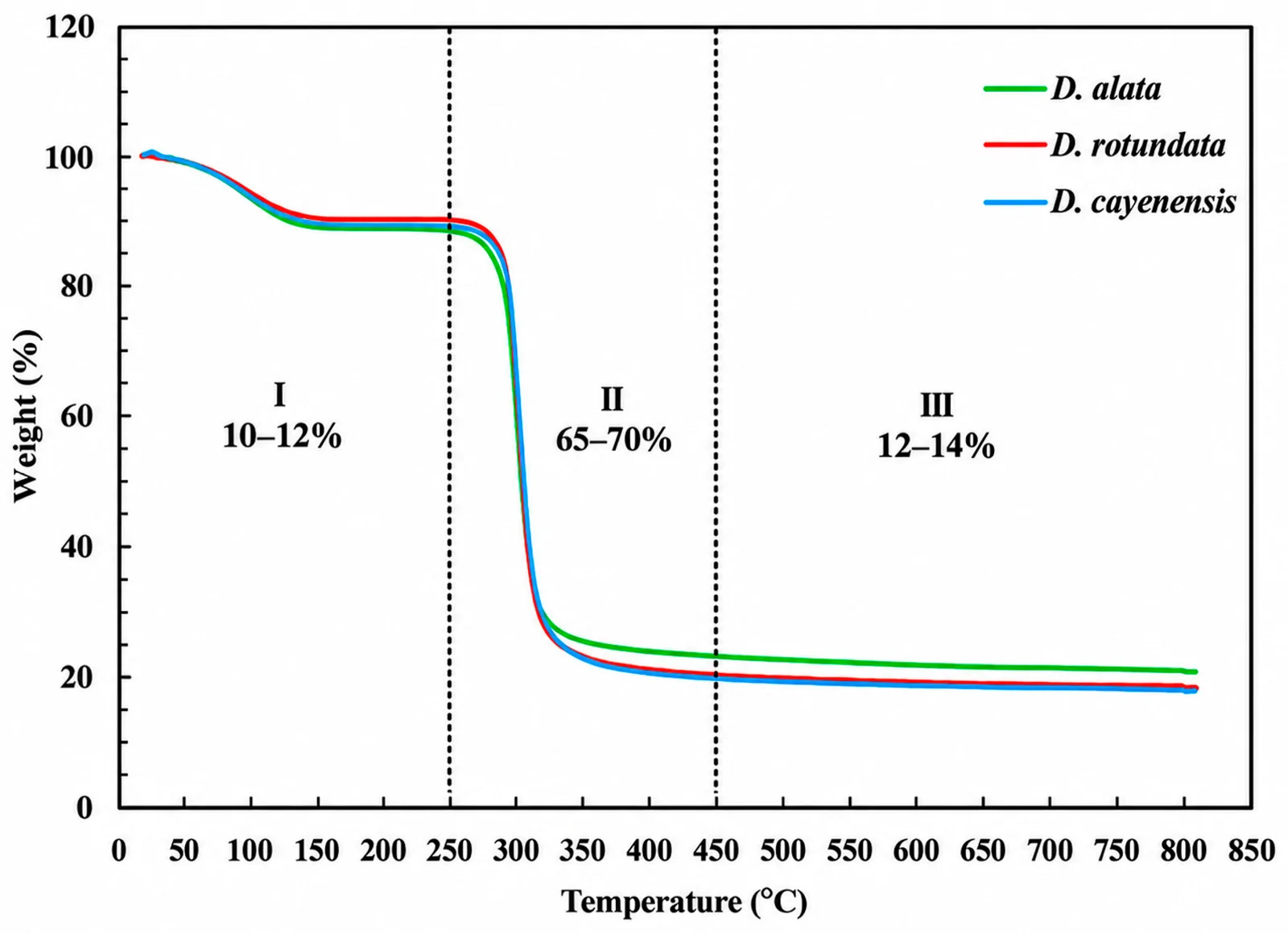
TGA curves and weight reduction of *D. alata*, *D. rotundata* and *D. cayenensis* yam starches.

Thermogravimetric analysis of the starches from *Dioscorea alata*, *Dioscorea rotundata*, and *Dioscorea cayenensis* revealed similar degradation profiles, characterized by three main stages of mass loss (Figure 4). The first stage, observed approximately between 50 and 250 °C, can be mainly associated with the removal of moisture, including adsorbed and weakly bound water, particularly at temperatures below approximately 150 °C. The mass loss observed at higher temperatures within this range may also involve the volatilization or thermal degradation of residual non-starch components, such as proteins or lipids. The initial mass loss is consistent with the hydrophilic nature of starch, whose hydroxyl groups favor the formation of hydrogen bonds with water molecules, a phenomenon previously reported for starches from various botanical sources [8,74].

The second stage, occurring at temperatures between approximately 250 and 450 °C, corresponds to the main thermal degradation of starch and accounts for the greatest loss of mass. In this temperature range, glycosidic bonds break and the amylose and amylopectin chains depolymerize, generating volatile compounds such as water, carbon dioxide, and other degradation products. This behavior has been reported for tuber and cereal starches [75,76,77]. The slight variations observed among species could be associated with differences in the structural composition of the starch. In this study, *D. rotundata* and *D. alata* had higher amylose contents (38.71% and 37.76%, respectively), while *D. cayenensis* had a lower content of this polysaccharide (32.58%) and a higher proportion of amylopectin (67.42%). The higher proportion of amylose could promote stronger intermolecular interactions, contributing to greater relative thermal stability, while the highly branched structure of amylopectin may facilitate thermal degradation processes.

Finally, at temperatures above 450 °C, a third stage is observed that is associated with carbonization and degradation of the carbonaceous residue formed during the decomposition of the polysaccharide. The final residue obtained at high temperatures can be attributed to carbonized material and the presence of small amounts of mineral compounds, a behavior consistent with that reported for yam and sweet potato starches analyzed by TGA [8].

### 3.5. Rheological Analysis

**Figure 5:**
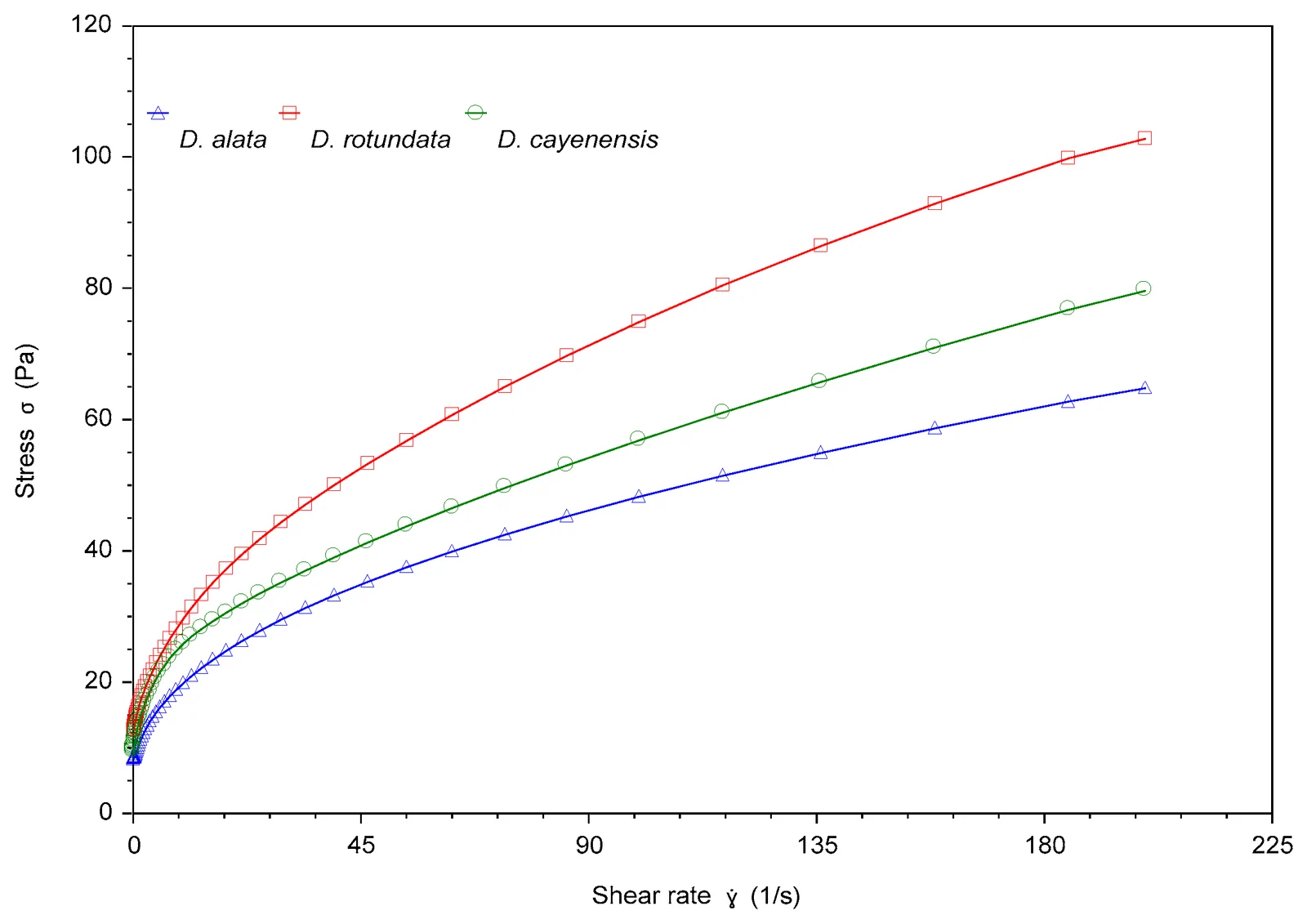
Flow curves for starches from *D. alata*, *D. rotundata* and *D. cayenensis*.

Figure 5 shows the flow curves of the starches from *D. alata*, *D. rotundata*, and *D. cayenensis*, while Table 7 presents the rheological parameters obtained using the Herschel–Bulkley model. The curves show that shear stress increases with shear rate, but in a nonlinear manner, indicating pseudoplastic non-Newtonian behavior. This behavior is characteristic of suspensions and gels of gelatinized starch, where viscosity decreases as shear rate increases due to the alignment and entanglement of the amylose and amylopectin polymer chains in the direction of flow [78,79,80].

The flow behavior index (*n*) obtained was less than 1 for all the starches analyzed (0.46–0.55), confirming the pseudoplastic behavior of the systems. Among the species evaluated, *D. rotundata* exhibited the highest *n* value, indicating slightly less pseudoplastic behavior compared to *D. alata* and *D. cayenensis*. Similar values have been reported by Ma et al. [80] and Ye et al. [77] in yam starch suspensions, where the *n* value typically ranges between 0.3 and 0.6 depending on concentration, molecular structure, and gelatinization conditions.

The consistency coefficient (*K*), which serves as an indirect measure of the system’s viscosity, was highest for *D. cayenensis*, followed by *D. alata* and *D. rotundata*. This parameter is directly related to the flow resistance of the starch gel and depends largely on structural factors such as amylose content, amylopectin chain length, and the degree of intermolecular interaction [39]. Ye et al. [77] reported that structural modifications in starch can significantly alter this parameter due to changes in interchain interactions and in the formation of three-dimensional networks during gelatinization.

The initial stress (*τ_0_*) showed significant differences among the studied species, being highest in *D. rotundata*, followed by *D. cayenensis* and *D. alata*. This parameter reflects the energy required to initiate flow in the system and is typically associated with the strength of the structural network formed by starch chains after gelatinization. A higher initial stress suggests the presence of a more resistant gel structure or greater interaction between amylose and amylopectin molecules [77,80].

The differences observed among species may be related to the structural composition of the starch, particularly to the relative content of amylose and amylopectin. In this study, *D. rotundata* had the highest amylose content, which could promote greater interaction between linear chains during gelatinization, contributing to the formation of more compact networks and increasing the force required to initiate flow. In contrast, *D. cayenensis* showed a higher amylopectin content, which may favor the formation of more viscous systems due to the greater swelling capacity and water retention of this highly branched fraction.

**Figure 6:**
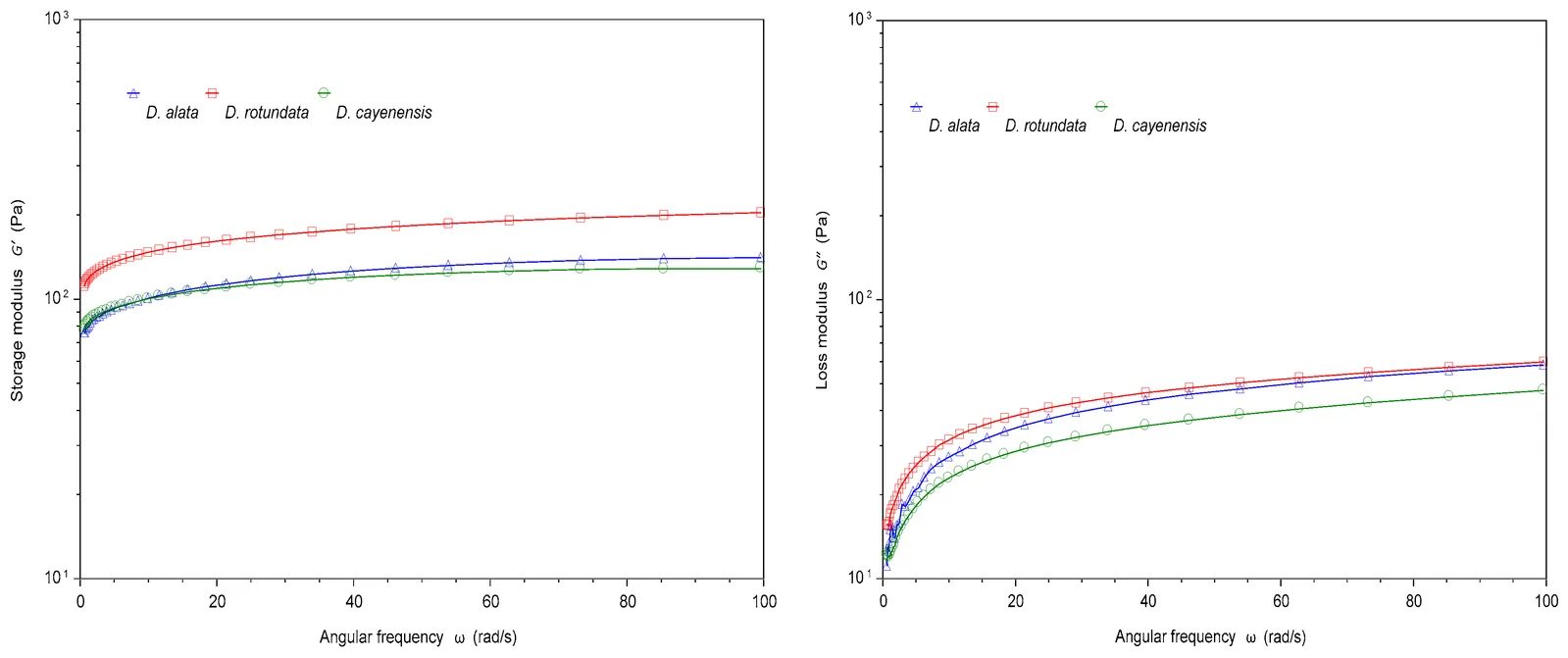
Storage modulus (G′) and loss modulus (G″) of the starches from *D. alata*, *D. rotundata* and *D. cayenensis*.

Furthermore, the viscoelastic behavior of yam starch pastes was evaluated using frequency sweeps, analyzing the evolution of the storage modulus (G′) and the loss modulus (G″) as a function of angular frequency (ω). As shown in Figure 6, for the three *Dioscorea* starches studied, the storage modulus was consistently higher than the loss modulus (G′ > G″) across the entire evaluated frequency range (0.1–100 rad s^−1^). This behavior indicates that the gelatinized dispersions exhibit a predominantly elastic response.

The predominance of G′ over G″ suggests the formation of a weak three-dimensional structural network arising primarily from the interaction between leached amylose molecules and swollen amylopectin regions originating from granules partially disintegrated during gelatinization [77]. This type of behavior was described by Zou et al. [39] for yam starches, where intermolecular interactions contribute to the formation of viscoelastic structures with a predominance of the elastic component.

In a cross-species comparison, the starch from *D. rotundata* exhibited the highest values for both G′ and G″ across the entire frequency range, indicating greater structural rigidity and a stronger viscoelastic network. This behavior suggests a greater capacity for interaction between polysaccharide chains, possibly associated with differences in molecular composition, particularly in amylose content, the length of the amylopectin chains, or the degree of granule swelling during gelatinization.

On the other hand, the starches from *D. alata* and *D. cayenensis* showed lower values for both moduli, with curves that were relatively close to each other. However, *D. alata* exhibited slightly higher G′ values compared to *D. cayenensis*, suggesting a more developed structural network. In all three systems, both G′ and G″ increased gradually with increasing angular frequency. This frequency dependence indicates that the formed structures exhibit viscoelastic characteristics dependent on deformation time, which is typical of weak gels formed by reversible physical interactions between macromolecules [77].

### 3.6. Pasting Properties

**Figure 7:**
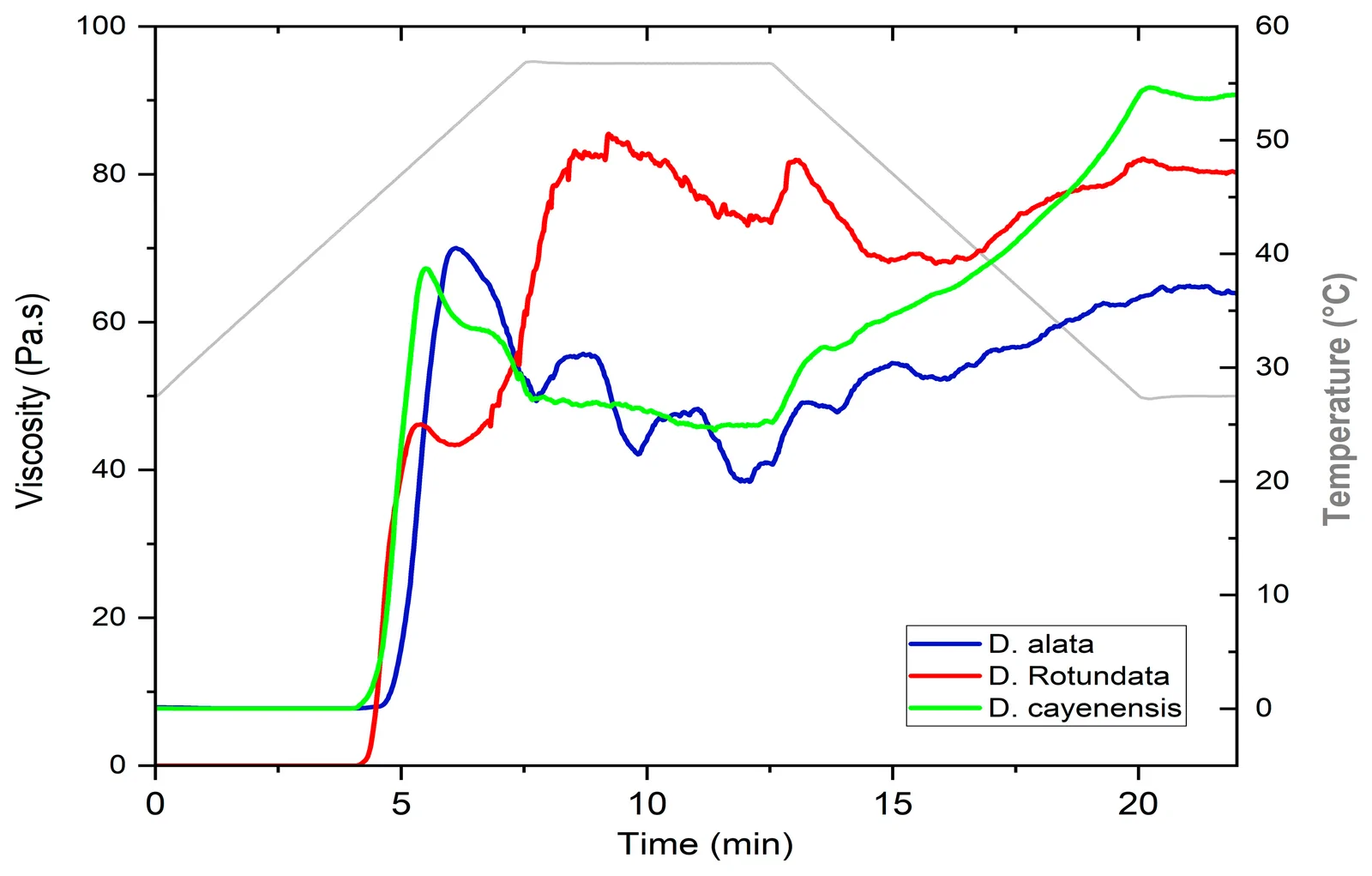
Pasting curves for the starches of *D. alata*, *D. rotundata* and *D. cayenensis*.

Table 8 and Figure 7 present the pasting parameters and viscosity profiles of the yam starches. The pasting temperature differed significantly among the samples. *D. alata* exhibited the highest pasting temperature (77.30), followed by *D. cayenensis* (75.07), whereas *D. rotundata* showed the lowest value (74.57). According to the statistical analysis, *D. alata* and *D. cayenensis* did not differ significantly from each other, but both exhibited significantly higher values than *D. rotundata*. A higher pasting temperature generally indicates greater resistance of starch granules to swelling and structural disruption during heating, which may be associated with stronger internal molecular interactions and greater structural organization within the granule [14].

Regarding peak viscosity, *D. rotundata* exhibited the highest value (44.92), followed by *D. alata* (39.36) and *D. cayenensis* (37.86). The latter two species did not differ significantly from each other, whereas *D. rotundata* showed a significantly higher peak viscosity. Peak viscosity reflects the extent of granule swelling and water uptake before the onset of granule disintegration and is influenced by the balance between swelling forces and internal cohesive forces within the starch granule [13,81]. Therefore, the higher peak viscosity observed for *D. rotundata* suggests a greater capacity for granule swelling during heating. This behavior may be related to its molecular composition and morphological characteristics, including amylose content, since higher amylose levels can restrict swelling, whereas lower amylose contents generally favor granule expansion [14,61]. The trough viscosity followed a similar trend, with *D. rotundata* showing the highest value (39.16), while *D. cayenensis* (25.12) and *D. alata* (21.61) exhibited significantly lower values and did not differ significantly from each other. Trough viscosity represents the minimum viscosity reached after peak viscosity during continued heating and agitation. Thus, the relatively high trough viscosity of *D. rotundata* suggests that its swollen granules retained a greater degree of structural integrity during the heating and shearing stage. In contrast, the lower trough viscosities observed for *D. alata* and *D. cayenensis* indicate a greater reduction in viscosity following the peak, reflecting differences in the stability of their swollen granules under thermal and mechanical stress [13].

These differences are also reflected in the breakdown viscosity, which was highest for *D. alata* (17.75), followed by *D. cayenensis* (12.73) and *D. rotundata* (5.75). *D. rotundata* showed a significantly lower breakdown value than *D. alata*, whereas *D. cayenensis* did not differ significantly from either species. Breakdown viscosity is commonly associated with the susceptibility of swollen starch granules to disintegration under heating and shear; therefore, lower values generally indicate greater paste stability [13]. In this regard, the low breakdown value of *D. rotundata* suggests greater resistance of its swollen granules to thermal and mechanical disruption. Conversely, the higher breakdown value observed in *D. alata* indicates greater susceptibility to granule disintegration during continued heating and agitation. This behavior may be related to differences in the strength of internal granular interactions and the extent of amylose leaching during heating [61,82].

Final viscosity showed a markedly different pattern from peak viscosity. *D. rotundata* exhibited the highest final viscosity (70.41), followed by *D. cayenensis* (49.46) and *D. alata* (41.33), with all three samples differing significantly from one another. Final viscosity reflects the ability of starch to form a viscous paste or gel-like structure during cooling, as starch chains reassociate and establish intermolecular interactions, including hydrogen bonding [13]. The substantially higher final viscosity of *D. rotundata* therefore indicates a greater capacity to develop viscosity during cooling compared with the other starches.

In contrast, setback viscosity did not show significant differences among the three yam starches, despite the numerical differences observed. *D. rotundata* presented the highest setback value (31.25), followed by *D. cayenensis* (24.34) and *D. alata* (19.72); however, all samples were statistically similar. Setback viscosity is generally associated with the reassociation of starch chains during cooling and is frequently used as an indicator of the tendency toward retrogradation [11,13]. Consequently, the numerical differences observed among the samples should not be interpreted as evidence of significantly different retrogradation tendencies. The absence of significant differences suggests that the three yam starches may exhibit comparable behavior regarding starch-chain reassociation under the conditions evaluated.

From a technological standpoint, these differences have important implications for the potential application of the starches in food systems. *D. rotundata* exhibited the highest peak, trough, and final viscosities, together with the lowest breakdown value, indicating a strong capacity for swelling, high paste development during cooling, and greater resistance to thermal and mechanical disruption. This profile may be advantageous in applications requiring high viscosity and paste stability, such as thickening systems and structured food products. *D. alata*, in contrast, showed the highest pasting temperature and breakdown viscosity, suggesting greater resistance to the onset of pasting but lower stability once the granules had swollen and were subjected to continued heating and shear. *D. cayenensis* generally displayed intermediate behavior, particularly in trough, final, and breakdown viscosities.

Although *D. rotundata* exhibited the numerically highest setback value, the lack of statistically significant differences among the three starches indicates that no clear differences in retrogradation tendency can be established based on this parameter alone. Therefore, the technological behavior of these starches appears to be more strongly differentiated by their swelling, pasting, and thermal stability characteristics than by their setback values. These differences may be relevant for selecting specific yam starches according to the viscosity, stability, and textural properties required in particular food applications [14,83].

## 4. Conclusions

The native starches from *Dioscorea alata*, *Dioscorea rotundata*, and *Dioscorea cayenensis* exhibited morphological, physicochemical, thermal, functional, binding, and rheological characteristics that demonstrate their potential as ingredients in food applications. The results showed variations in amylose content and in functional properties such as water and oil retention capacity, swelling power, and solubility, with *D. cayenensis* standing out for its greater swelling capacity at high temperatures. Dough-forming properties revealed significant differences among the species, with *D. rotundata* exhibiting higher peak viscosity and greater thermal and shear stability, suggesting greater resistance during thermal processing and potential to improve moisture retention in baked goods.

For its part, *D. alata* exhibited a higher pasting temperature and breakdown viscosity, indicating greater resistance to the onset of pasting but greater susceptibility to structural disruption during continued heating and shear. *D. rotundata* exhibited the highest final viscosity, while setback viscosity did not differ significantly among the species. Likewise, the pseudoplastic rheological behavior and the predominance of the elastic modulus over the viscous modulus indicate the formation of viscoelastic systems with good structural stability, with *D. rotundata* exhibiting the highest flow resistance and greater structural rigidity, as indicated by its higher yield stress (τ_0_) and G′ and G″ values. Taken together, these properties suggest that the studied starches have high potential as structuring and functional agents in baking, where they can contribute to the texture, stability, and quality of the final product. However, the application of physical, chemical, or enzymatic modifications is recommended to optimize their technological performance, particularly in reducing retrogradation and improving the shelf life of baked goods.

## Figures and Tables

**Table 1:** Particle size distribution of yam starches.

Parameter/Sample	*D. alata*	*D. rotundata*	*D. cayenensis*
D [10] μm	18.55 ± 0.23 ^b^	16.76 ± 0.16 ^c^	19.30 ± 0.32 ^a^
D [50] μm	26.98 ± 0.07 ^b^	24.15 ± 0.10 ^c^	29.03 ± 0.28 ^a^
D [90] μm	38.70 ± 0.51 ^b^	34.36 ± 0.10 ^c^	43.16 ± 0.54 ^a^
D [4;3] μm	27.86 ± 0.12 ^b^	24.91 ± 0.09 ^c^	30.26 ± 0.28 ^a^
D [3;2] μm	25.86 ± 0.05 ^b^	23.20 ± 0.15 ^c^	27.66 ± 0.33 ^a^

Means with the same letter in the same row are not significantly different (*p* < 0.05).

**Table 2:** pH, titratable acidity and color of yam starches.

Analysis/Sample	*D. alata*	*D. rotundata*	*D. cayenensis*
pH	6.07 ± 0.18 ^b^	6.82 ± 0.05 ^a^	7.10 ± 0.06 ^a^
Acidity	0.31 ± 0.07 ^a^	0.07 ± 0.01 ^b^	0.05 ± 0.07 ^b^
L*	91.49 ± 0.04 ^c^	92.95 ± 0.03 ^b^	94.09 ± 0.02 ^a^
a*	0.68 ± 0.01 ^a^	0.06 ± 0.02 ^b^	−0.76 ± 0.03 ^c^
b*	4.16 ± 0.04 ^a^	3.48 ± 0.07 ^b^	3.46 ± 0.02 ^b^
%WI	90.50 ± 0.02 ^c^	92.13 ± 0.02 ^b^	93.11 ± 0.02 ^a^

Means with the same letter in the same row are not significantly different (*p* < 0.05).

**Table 3:** Proximate composition, and amylose and amylopectin content of yam starches.

Analysis/Sample	*D. alata*	*D. rotundata*	*D. cayenensis*
Moisture (%)	11.99 ± 0.38 ^a^	6.19 ± 0.59 ^c^	8.83 ± 0.52 ^b^
Crude Fat (%)	0.01 ± 0.01 ^a^	0.02 ± 0.01 ^a^	0.03 ± 0.02 ^a^
Crude fiber	<1.0 g/100 g	<1.0 g/100 g	<1.0 g/100 g
Ashes (%)	0.31 ± 0.05 ^a^	0.12 ± 0.02 ^a^	0.20 ± 0.10 ^a^
Protein (%)	0.96 ± 0.09 ^a^	0.46 ± 0.05 ^b^	0.37 ± 0.05 ^b^
Carbohydrates (%)	86.73 ± 0.30 ^c^	93.33 ± 0.55 ^a^	90.57 ± 0.90 ^b^
Amylose (g/100 g)	37.76 ± 0.34 ^b^	38.71 ± 0.25 ^a^	32.58 ± 0.31 ^c^
Amylopectin (g/100 g)	62.24 ± 0.34 ^b^	61.29 ± 0.25 ^c^	67.42 ± 0.31 ^a^

Means with the same letter in the same row are not significantly different (*p* < 0.05).

**Table 4:** Functional properties of yam starches.

Analysis/Sample	*D. alata*	*D. rotundata*	*D. cayenensis*
WHC gg	2.15 ± 0.02 ^a^	2.03 ± 0.02 ^b^	1.97 ± 0.04 ^b^
OHC mLg	0.83 ± 0.04 ^a^	0.52 ± 0.03 ^c^	0.699 ± 0.04 ^b^

Means with the same letter in the same row are not significantly different (*p* < 0.05).

**Table 5:** Swelling power and solubility of yam starches.

Temperature (°C)	*D. alata*		*D. rotundata*		*D. cayenensis*	
	PO	SO	PO	SO	PO	SO
55	2.25 ± 0.11 ^a^	0.00 ± 0.00 ^a^	1.93 ± 0.06 ^ab^	0.00 ± 0.00 ^a^	1.75 ± 0.20 ^b^	0.00 ± 0.00 ^a^
65	2.41 ± 0.04 ^a^	1.33 ± 0.10 ^ab^	1.90 ± 0.09 ^b^	1.88 ± 0.50 ^a^	1.93 ± 0.19 ^b^	0.98 ± 0.12 ^b^
75	2.26 ± 0.06 ^a^	0.40 ± 0.08 ^a^	2.00 ± 0.04 ^a^	1.25 ± 0.85 ^a^	2.20 ± 0.10 ^a^	0.65 ± 0.50 ^a^
85	8.08 ± 0.05 ^a^	4.78 ± 0.07 ^a^	2.13 ± 0.20 ^b^	1.98 ± 0.20 ^c^	7.83 ± 0.45 ^a^	3.83 ± 0.52 ^b^
95	10.61 ± 0.39 ^ab^	5.13 ± 0.72 ^a^	9.99 ± 0.14 ^b^	2.68 ± 0.75 ^b^	11.0 ± 0.14 ^a^	2.86 ± 0.50 ^b^

Means with the same letter in the same row are not significantly different (*p* < 0.05).

**Table 6:** DSC of starches from *D. alata*, *D. rotundata* and *D. cayenensis*.

Analysis/Sample	*D. alata*	*D. rotundata*	*D. cayenensis*
T_o_ (°C)	76.69 ± 0.03 ^a^	73.53 ± 0.01 ^b^	73.70 ± 0.09 ^b^
T_p_ (°C)	79.22 ± 0.13 ^a^	78.07 ± 0.40 ^b^	79.74 ± 0.06 ^a^
T_c_ (°C)	82.34 ± 0.32 ^b^	82.49 ± 0.79 ^ab^	84.41 ± 0.06 ^a^
ΔH (J/g)	2.42 ± 0.11 ^a^	2.83 ± 0.52 ^a^	3.50 ± 0.38 ^a^

Means with the same letter in the same row are not significantly different (*p* < 0.05).

**Table 7:** Rheological parameters.

Analysis/Sample	*D. alata*	*D. rotundata*	*D. cayenensis*
*τ_0_* (*Pa*)	4.77 ± 0.14 ^c^	9.82 ± 0.33 ^a^	7.97 ± 0.38 ^b^
*K* (*Pa·s^n^*)	5.43 ± 1.36 ^a^	5.02 ± 0.74 ^a^	5.97 ± 0.43 ^a^
Index *n*	0.46 ± 0.02 ^b^	0.55 ± 0.03 ^a^	0.47 ± 0.01 ^b^

Means with the same letter in the same row are not significantly different (*p* < 0.05).

**Table 8:** Pasting properties.

Analysis/Sample	*D. alata*	*D. rotundata*	*D. cayenensis*
Pasting temperature (°C)	77.30 ± 0.50 ^a^	74.57 ± 1.03 ^b^	75.07 ± 0.53 ^b^
Peak viscosity (Pa.s)	39.36 ± 1.08 ^b^	44.92 ± 1.47 ^a^	37.86 ± 1.45 ^b^
Trough viscosity (Pa.s)	21.61 ± 1.44 ^b^	39.16 ± 8.81 ^a^	25.12 ± 0.57 ^b^
Final viscosity (Pa.s)	41.33 ± 6.98 ^b^	70.41 ± 9.39 ^a^	49.46 ± 8.49 ^b^
Breakdown	17.75 ± 2.51 ^a^	5.75 ± 7.49 ^b^	12.73 ± 1.69 ^ab^
Setback	19.72 ± 6.04 ^a^	31.25 ± 6.29 ^a^	24.34 ± 8.90 ^a^

Means with the same letter in the same row are not significantly different (*p* < 0.05).

## Data Availability

The manuscript incorporates all data sets produced or examined throughout this research study.
